# Blisters and Milia around the Peritoneal Dialysis Catheter: A Case of Localized Bullous Pemphigoid

**DOI:** 10.3390/dermatopathology9030033

**Published:** 2022-08-04

**Authors:** Andrea Michelerio, Carlo Tomasini

**Affiliations:** 1 Dermatology Clinic, Department of Clinical-Surgical, Diagnostic and Pediatric Sciences, University of Pavia, 27100 Pavia, Italy; 2 Dermatology Clinic, Fondazione IRCCS Policlinico San Matteo, 27100 Pavia, Italy

**Keywords:** bullous pemphigoid, bullous dermatoses, dialysis, peritoneal dialysis

## Abstract

We report on the appearance of multiple tense blisters surrounding the exit site of a Tenckhoff catheter in a 79-year-old woman with end-stage renal disease in peritoneal dialysis. The differential diagnoses included a contact allergic or irritative dermatitis to peritoneal dialysis catheter material and antiseptic agents, bacterial infection, and herpes virus infection, but milia were a clue for a subepidermal blistering disease and lead to appropriate investigations. The laboratory findings, the histopathological examination and the direct immunofluorescence assay confirmed the diagnosis of localized bullous pemphigoid. The disorder typically occurs in elderly people and may be related to drugs, hematological malignancies or neurological conditions but it can also be a complication of hemodialysis or peritoneal dialysis.

## 1. Introduction

Bullous pemphigoid (BP) is mostly thought of as a generalized blistering eruption affecting the trunk and the proximal extremities. Although this is a correct picture of the disease, a number of patients with BP may have localizedcutaneous disease either persisting over a variable period of time or initially localized and followed by dissemination later on in the clinical course [1]. We report on the appearance of multiple tense blisters surrounding the exit site of a Tenckhoff catheter in a 79-year-old woman with end-stage renal disease in peritoneal dialysis. The laboratory findings, the histopathological examination and the direct immunofluorescence assay confirmed the diagnosis of localized bullous pemphigoid. The disorder typically occurs in elderly people and may be related to drugs, hematological malignancies, or neurological condition but it can also be a complication of hemodialysis or peritoneal dialysis

## 2. Case Report

**Figure 1:**
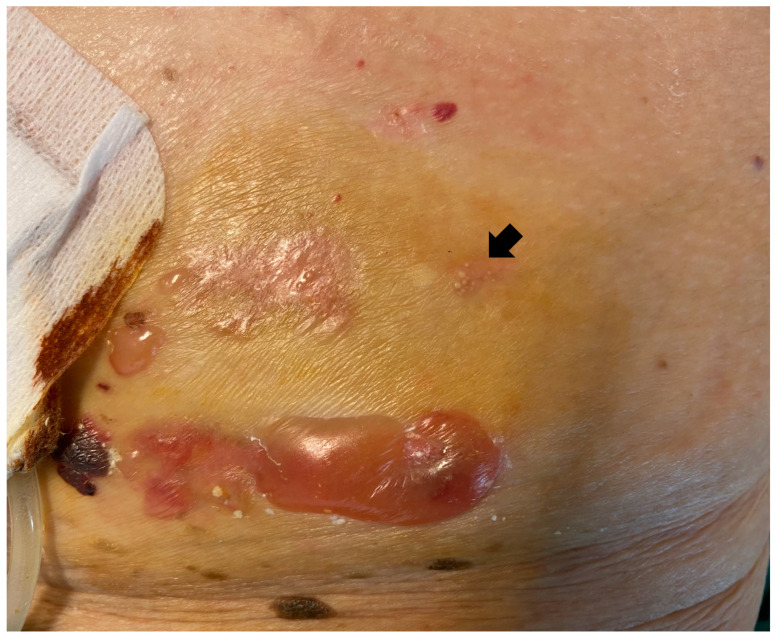
Tense vesicles, blisters and crusts surrounding around the peritoneal dialysis catheter exit site. Milia can be observed (arrow).

A 79-year-old woman with a previous medical history of hypertension, hypercholesterolemia, gout and end-stage renal disease presented to our Clinic with a 6-month history of a worsening pruritic vesico-bullous rash around her peritoneal dialysis (PD) catheter exit site. On examination, there were tense blisters with sero-hemorrhagic content over non-erythematous skin (Figure 1). Crops of pinpoint milia (Figure 1, arrow) were also observed. The oral mucosa was spared, and the rest of the skin was normal. The patient was apyretic and her medication list included simvastatin, recombinant human erythropoietin, vitamin D supplement, amlodipine, atenolol, pantoprazole and febuxostat. Laboratory findings showed increased ESR (68 mm/h), mild eosinophilia (0.58 × 10^3^/µL, 6.9%), a high IgE value (520.4 IU/mL) and normal RCP. Despite the change in the products used for the catheter care and dressings, as suggested by the dialysis service, the lesions had not improved. No bacteria or fungi were isolated from repeated cultures of the PD exit site and bullous lesions. Milia led us to suspect a subepidermal blistering disease.

**Figure 2:**
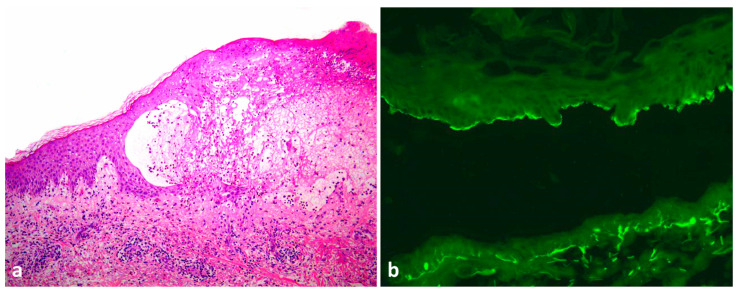
Punch biopsy of the lesion revealing a subepidermal blister containing fibrin, eosinophils and mononuclear cells, consistent with bullous pemphigoid (**a**). Direct immunofluorescence on salt-split skin reveals IgG on the epidermal side of split skin (blister roof) (**b**).

A skin biopsy revealed focal epidermal spongiosis, subepidermal clefts and a predominantly eosinophilic dermal infiltrate (Figure 2a). Salt-split direct immunofluorescence of the perilesional skin disclosed continuous linear deposits of C3 and immunoglobulin G on the roof of the split (Figure 2b). Enzyme-linked immunosorbent assay showed a small increase in circulating antibodies to BP180 (16 U/mL, normal values < 9 U/mL). A diagnosis of localized bullous pemphigoid was made.

**Figure 3:**
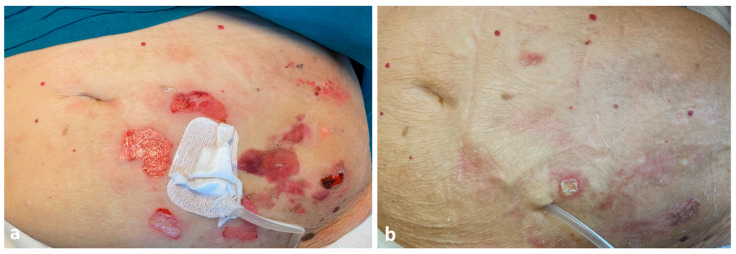
Healing erosions without new blisters around the peritoneal dialysis catheter exit site after two weeks of therapy (**a**). Resolved/healed skin lesions six weeks later (**b**).

The patient was treated with a two-week course of low-dose prednisone therapy (0.5 mg/kg/die) and topical 0.05% clobetasol propionate ointment once daily, with resolution of the lesions and residual slight hyperpigmentation (Figure 3a,b). Dapsone 25 mg daily and clobetasol propionate ointment 0.05% twice weekly were started as a maintenance therapy. No recurrence of peristomal bullae or onset of cutaneous lesions in other skin sites was observed at 6-month follow-up and the patient did not require the removal of the PD catheter.

## 3. Discussion

Bullous pemphigoid (BP) is an autoimmune blistering disease most commonly arising in elderly individuals. The classical clinical findings are widespread, tense, fluid-filled blisters on an erythematous or non-inflammatory base. The disease may start as a localized eruption in 15% to 30% of patients [2] although strictly localized forms are much less frequent, accounting for 2.5% of cases [1]. The majority of localized cases have a predilection for the lower extremities [2], but lesions in sites of prior radiation, lymphedema, surgical wounds, colostomy or urostomy stoma have rarely been described [3]. Dyshidrosiform pemphigoid localized on palmoplantar areas is also a rare presentation [4].

The onset of BP in patients receiving hemodialysis has been reported [5,6,7,8,9,10,11], even in young patients [8,9,10], but the real incidence is unknown. In a few patients with an arteriovenous fistula (AVF) for hemodialysis access, the blisters have developed on the skin adjacent to the AVF [5,7,8,9,10], but BP lesions developed around PD catheter in only one case [11], as in our case.

The damaged epithelium at the site of chronic irritation or contact dermatitis [12], an inflammatory reaction to a bacterial colonization [13] and/or the activation of the immune system with increased capillary permeability are possible trigger factors of localized BP, contributing to the dermal–epidermal junction antigens exposure. In an immunologically susceptible individual this might have led to the loss of immune tolerance and to the antibody production. No drug with a causative link with BP, such as aldosterone antagonists, dipeptidyl peptidase 4 inhibitors, anticholinergics, and dopaminergic medications was present in our patient’s anamnesis [14,15,16].

The differential diagnosis of localized BP at the PD catheter exit site mainly includes contact allergic or irritative dermatitis to PD catheter material [17], antibiotic preparations (mupirocin and polysporin) [18] and antiseptic agents [19], bacterial infection, and herpes virus infection. The diagnosis of contact allergic dermatitis is usually established on clinical grounds based on the characteristic appearance of rash, negative Gram stain and culture of the PD exit site and favorable response to withdrawal of the suspected agent along with performing patch tests.

Bacterial infection (mostly due to Staphylococcus aureus and Pseudomonas aeruginosa) at the PD exit site typically appears with purulent and/or bloody drainage from the PD catheter exit site, with surrounding erythema, tenderness, and swelling. Bullous impetigo is more common in children and histologically the blisters are subcorneal. Prompt diagnosis with isolation of the pathogen by culture and treatment are essential to prevent sepsis and PD-associated peritonitis [20]. A polymerase chain reaction for herpes simplex virus 1 and 2 DNA excludes a peristomal herpetic infection.

Epidermolysis bullosa acquisita (EBA) may be considered in the differential diagnosis of our case of peristomal BP as it shows a predilection for trauma sites and the tendency to heal with milia. Secondary milia are benign keratinous cysts that result from the regeneration process of sweat glands or hair follicles and may appear on sites of previous blistering [21]. The exact etiology of multiple milia formation in subepidermal blistering disorders is still unknown, but they are believed to develop in the process of regenerating sweat glands or hair follicles damaged in the blistering [22], possibly due to aberrant interaction between the hemidesmosomes and the extracellular matrix components beneath the hemidesmosomes [23]. Evaluation using the salt-split technique and anti-type VII collagen IgG determination might prove useful in the differential diagnosis of EBA and BP.

## 4. Conclusions

In conclusion, this case documents the second case of localized BP around the peritoneal dialysis catheter exit site. Current estimates suggest that approximately 11% of the global dialysis population patients receive peritoneal dialysis, with an annual global growth rate of 8% [24]. Thus, an increase in this presentation of localized BP can be expected. The clinical manifestation might mimic a contact dermatitis, a more common and benign dermatologic condition, a bacterial infection, which is a serious and potentially fatal event, or other conditions, delaying a proper diagnosis and treatment. In general, localized BP has a more benign disease course because it responds better to local treatments than the generalized form [25]. Moreover, the removal of the PD catheter was not required in our case or in the one described by Giunzioni [11]. Nevertheless, the localized disease may precede a diffuse involvement and a regular follow-up is necessary [25].

## Data Availability

Data are contained within the article.
